# Sustainability in surgical practice: a collaborative call toward environmental sustainability in operating rooms

**DOI:** 10.1007/s00464-024-10962-0

**Published:** 2024-07-01

**Authors:** Shaneeta M. Johnson, Stefania Marconi, Manuel Sanchez-Casalongue, Nader Francis, Bright Huo, Adnan Alseidi, Yewande R. Alimi, Andrea Pietrabissa, Alberto Arezzo, Maximos Frountzas, Vittoria Bellato, Oleksii Potapov, Paul Barach, Miran Rems, Ricardo J. Bello, Sheetal Nijhawan, Wendelyn M. Oslock, Tejas S. Sathe, Ryan P. Hall, Benjamin Miller, Sarah Samreen, Jimmy Chung, Nana Marfo, Robert B. Lim, Jonathan Vandeberg, Myrthe M. Eussen, Nicole D. Bouvy, Patricia Sylla

**Affiliations:** 1 Department of Surgery, Morehouse School of Medicine, 720 Westview Drive, Atlanta, GA 30310 USA; 2 Satcher Health Leadership Institute, Morehouse School of Medicine, Atlanta, GA USA; 3 Department of Civil Engineering and Architecture, University of Pavia, Pavia, Italy; 4 IRCCS Policlinico San Matteo Foundation, Pavia, Italy; 5 Department of Surgery, Clinica San Camilo, Buenos Aires, Argentina; 6 Griffin Institute, London, UK; 7 Department of General Surgery, McMaster University, Ontario, CA USA; 8 Department of Surgery, University of California San Francisco, San Francisco, USA; 9 Department of Surgery, Medstar Georgetown University Hospital, Washington, DC USA; 10 Department of General Surgery, University of Pavia, Pavia, Italy; 11 Department of Surgical Sciences, University of Turin, Turin, Italy; 12 First Propaedeutic Department of Surgery, National and Kapodistrian University of Athens, Hippocration General Hospital, Athens, Greece; 13 Department of Minimally Invasive Surgery, University Hospital of Rome Tor Vergata, Rome, Italy; 14 Department of General Surgery, College of Medicine, University of Rzeszow, Rzeszow, Poland; 15 Thomas Jefferson University School of Medicine, Philadelphia, USA; 16 Department of General Surgery, Imperial College London, London, UK; 17 Department of General and Abdominal Surgery, General Hospital Jesenice, Jesenice, Slovenia; 18 Department of Surgery, Medical College of Wisconsin, Milwaukee, USA; 19 Department of Surgery, Sharon Regional Medical Center, Sharon, PA USA; 20 Department of Surgery, University of Alabama Birmingham, Birmingham, AL USA; 21 Department of Quality, Birmingham Veterans Affairs Medical Center, Birmingham, AL USA; 22 Department of Surgery, Tufts Medical Center, Boston, MA USA; 23 Cleveland Clinic Foundation, Cleveland, OH USA; 24 Division of Minimally Invasive Surgery, University of Texas Medical Branch, Galveston, TX USA; 25 Adventus Health Partners, Cincinnati, OH USA; 26 Ross University School of Medicine, Miramar, FL USA; 27 Department of Surgery, Wake Forest University, Atrium Carolinas Medical Center, Charlotte, NC USA; 28 Department of Surgery, Mayo Clinic Florida, Jacksonville, FL USA; 29 Department of Surgery, Maastricht University Medical Center, Maastricht, The Netherlands; 30 NUTRIM School of Nutrition and Translational Research in Metabolism, Maastricht University, Maastricht, The Netherlands; 31 Division of Colon and Rectal Surgery, Mount Sinai Health System, New York, NY USA

**Keywords:** Surgical sustainability, Environmental sustainability, Surgical practice, Carbon footprint, Greenhouse gas emissions, Green operating rooms, Climate change, Decarbonization, Minimally invasive surgery

## Abstract

**Background:**

The healthcare system plays a pivotal role in environmental sustainability, and the operating room (OR) significantly contributes to its overall carbon footprint. In response to this critical challenge, leading medical societies, government bodies, regulatory agencies, and industry stakeholders are taking measures to address healthcare sustainability and its impact on climate change. Healthcare now represents almost 20% of the US national economy and 8.5% of US carbon emissions. Internationally, healthcare represents 5% of global carbon emissions. US Healthcare is an outlier in both per capita cost, and per capita greenhouse gas emission, with almost twice per capita emissions compared to every other country in the world.

**Methods:**

The Society of American Gastrointestinal and Endoscopic Surgeons (SAGES) and the European Association for Endoscopic Surgery (EAES) established the Sustainability in Surgical Practice joint task force in 2023. This collaborative effort aims to actively promote education, mitigation, and innovation, steering surgical practices toward a more sustainable future.

**Results:**

Several key initiatives have included a survey of members' knowledge and awareness, a scoping review of terminology, metrics, and initiatives, and deep engagement of key stakeholders.

**Discussion:**

This position paper serves as a Call to Action, proposing a series of actions to catalyze and accelerate the surgical sustainability leadership needed to respond effectively to climate change, and to lead the societal transformation towards health that our times demand.

## The role and purpose of the sustainability in surgical practice (SSP) task force

Climate change is a global concern that poses an existential threat to human health [1]. Sustainability impacts the well-being of individuals, families, and communities worldwide. Unfortunately, the healthcare industry contributes significantly to the global carbon footprint and is responsible for almost 5% of greenhouse gas emissions [2]. In the United States alone, the sector accounts for 8.5% of national greenhouse gas emissions [3]. Urgent action is needed to mitigate these impacts. The operating room significantly contributes to healthcare facilities' sustainability impact via its significant waste generation and energy consumption.

Professional medical societies have an ethical and social responsibility to model and inspire needed changes in human activity that will lead to a healthier world. We must raise awareness of the climate crisis, educate our members on the social and environmental impacts of the healthcare sector, and disseminate, and hold our colleagues to account related to, best practices on how to reduce their carbon footprint in health organizations. In December 2019, the European Commission launched the European Green Deal, which should help make Europe the first climate-neutral continent in the world. In 2021, the European Union climate legislation was adopted, binding the European Union to achieve climate neutrality by 2050 and setting a target of reducing net greenhouse gas emissions by at least 55% by 2030 as compared to 1990 levels [4]. Similarly, in 2022, in light of the fact that the health sector accounts for 8.5% of US GHG emissions, the US Department of Health and Human Services (HHS) announced the White House-HHS Health Sector Climate Pledge to reduce emissions by 50% by 2030 and achieve net zero emissions by 2050 [5]. The document was signed by hundreds of organizations, including hospitals, suppliers, insurance companies, group purchasing organizations, pharmaceutical and device companies, as well as professional medical and surgical societies, including SAGES (Health Sector Pledge | HHS.gov) [5]. The US National Academy of Medicine partnered with the federal government and leading healthcare sector entities to propose a Grand Challenge on Decarbonizing the US Health System [6].

**Fig. 1:**
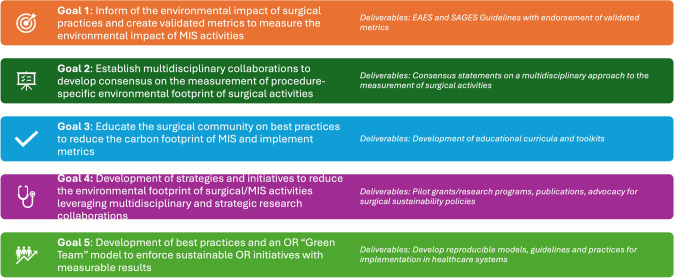
Goals of the SSP Task Force

In response to this, and in an acknowledgment of the vital role that both EAES and SAGES societies can contribute to all aspects of sustainability and surgical quality, the Sustainability in Surgical Practice (SSP) Taskforce has been created [7]. The main goals of this alliance are to educate surgeons and their teams and collaborate with strategic partners on the shared goal of implementing and sustaining strategies to reduce the environmental footprint of surgical practice, particularly in the field of minimally invasive surgery (Fig. 1).

## Enhancing sustainability in surgical practices—SSP key strategies and initiatives

Recognizing the health and other implications of sustainability and the crucial role of surgeons, surgical teams, and surgical practices in promoting sustainability, the SSP task force began the implementation of initiatives focusing on sustainability core competencies including:

- Knowledge: Developing educational programs, identifying the scope of knowledge gaps, Understanding the scope of the global surgical knowledge base, understanding current initiatives in surgical sustainability
- Skills-building: Building the skills repertoire needed to effect behavioral and attitude change, collaborating with key stakeholders to develop an understanding of the initiatives, barriers, and challenges to adopting a greener OR.
- Leadership: Implementing SAGES/EAES focus on the importance of surgical sustainability leadership for society resilience and impact in surgical practices [8].

These key SSP initiatives have included:

### SAGES and EAES membership survey

The SSP Taskforce surveyed the SAGES and EAES membership to assess sustainability knowledge and awareness. The survey covered several aspects, including attitudes, current knowledge, concerns, perceived barriers, and willingness to implement climate sustainability changes. The survey received 1024 complete responses from a global cohort of surgeons. It is the largest and first international assessment of surgeons' attitudes and understanding of carbon emissions generated from surgical activity and strategies to improve sustainability. The results demonstrated that many surgeons lack awareness about carbon footprint and sustainability. Although most surgeons (63% of respondents) viewed sustainability as a critical problem and were motivated to change their practices to improve it, less than half (43% of respondents) believed that climate change was a critical problem for the health of their patients. Additionally, only a minority of surgeons could estimate the carbon footprint of a surgical procedure (7.7% of respondents) or surgical supplies (6.5% of respondents), indicating a knowledge gap. The survey also highlights the need for targeted educational initiatives to address these knowledge gaps. Most respondents preferred less time-consuming educational modalities such as online webinars, online modules, and lectures. The most popular topics for education were waste management, supply chains, and reference cards.

### Comprehensive scoping review

We conducted a comprehensive scoping review was undertaken to better understand the terminology and outcome measures used in the field of surgical sustainability, delineate the various domains in gastrointestinal surgical practice that can impact the environment and review the initiatives with positive impacts on the environment. The key findings include:

I. Surgical waste is a major contributor to the carbon footprint. Waste reduction strategies focused on refusing, reducing, and recycling to limit waste production in operating rooms. These strategies include using reusable equipment, leaner equipment trays, and avoiding unnecessary use of equipment on surgical trays [9–13]. Studies have shown that a significant proportion of the surgical waste generated during routine surgeries, such as inguinal hernias, can be avoided, resulting in cost savings and reduced greenhouse gas emissions [14]. Additionally, research suggests that around 75% of equipment-related consumables can be reduced in common procedures like an appendectomy, which can result in significant cost savings and reduced environmental impact [15, 16]. A life cycle assessment (LCA) demonstrated that reusable equipment has a lower environmental impact than disposable options. This includes climate change potential, ecosystem quality, health impacts, and resource depletion [17]. The combined approach of educating staff to reduce waste and improve waste sorting and recycling had among the largest reported impact on reducing surgical footprint and in improving the climate [18, 19].
II. Anesthetic gases, especially desflurane, have a significant impact on carbon footprint during gastrointestinal surgery. Substituting alternative gases, local anesthetics, or intravenous anesthesia can lead to substantial environmental, financial, and social benefits and contribute to mitigating climate change and promoting sustainable practices in surgical procedures [20]. The use of anesthetic machines that limit the flow of gas can yield among one of the largest reported reductions in climate impact via reductions in surgical waste and carbon footprint [21].
III. The type of surgical approach impacts the environmental footprint. Minimally invasive procedures that use non-energized dissection techniques can reduce CO2 emissions [22]. However, the LCA analyses of robotic approaches reveal a higher energy usage than that of open surgical techniques. Robotic approaches in hernia repair have been associated with increased solid waste production, contributing to adverse downstream environmental impacts [23].
IV. Surgical complications may be associated with an increased carbon footprint. Anastomotic leaks, a common complication, leading to much morbidity and increased costs, can result in a substantially increased climate footprint per patient, including increased carbon dioxide equivalent (CO2eq) emissions, water consumption, and waste generation [24].
V. Simple and low-cost initiatives can reduce energy and water consumption in the OR. These include heat-recovery systems and judicious use of water while scrubbing or the use of alcohol-based scrubs or scrub-less alternatives [25].
VI. Educational and sustainability initiatives are essential to mitigate the environmental footprint of surgical practice. These initiatives include quantifying the utilization and costs associated with the use of reusable versus disposable surgical equipment, providing monthly feedback in the form of a “sustainability report card” on utilization and costs of disposable and reusable surgical supplies on the cost savings and waste reduction in the OR, implementing waste-reduction strategies, educating staff on proper waste sorting, and developing educational change management programs focused on surgical sustainability competencies and fellowships [26–28]. Education surrounding the mitigation of anesthetic gas use, particularly desflurane, was found to be significantly impactful in reducing the climate impact of surgical care and required close collaboration with anesthesia and surgical services leadership [21, 29, 30]
VII. Life cycle assessments are considered the gold standard in sustainability measures however they are resource-intensive and may be impractical in certain settings. The 10R model of circular economy is an effective method to address sustainability measures.

### Industry and key stakeholder engagement

**Fig. 2:**
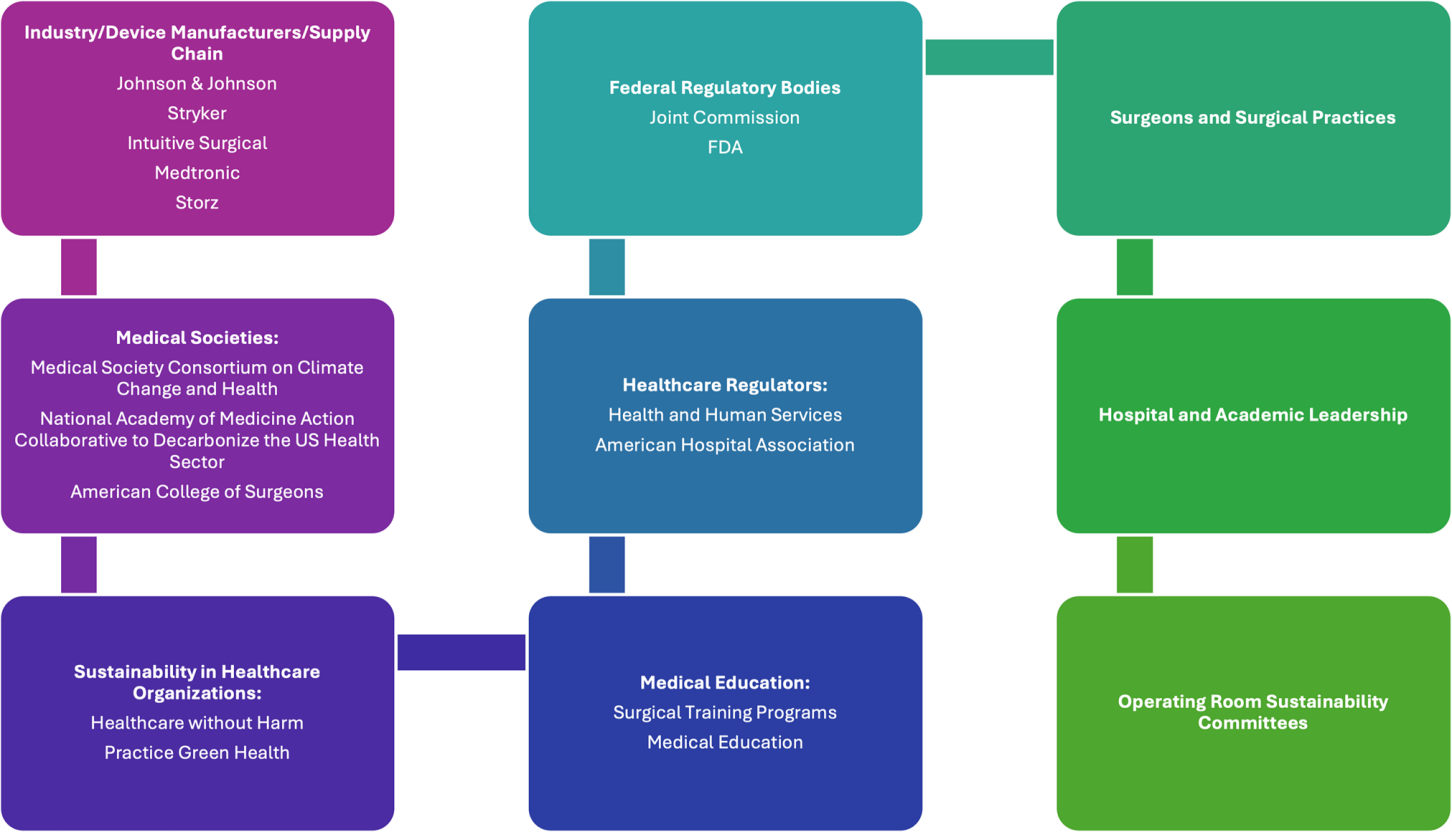
SSP ongoing and planned collaboration with key stakeholders

A series of targeted meetings were conducted with key industry stakeholders to assess their knowledge, interests, initiatives, and barriers related to sustainability in surgical practices. These meetings highlighted that several device manufacturers are actively involved in sustainability initiatives. Targeted interventions include packaging redesign, recycling, reuse, and measuring the carbon footprint of their manufacturing processes. However, industry leads identified obstacles to implementing initiatives including restrictions regarding the regulatory process around product redesign both in the US and in Europe. The insights gained from these meetings provided a basis for understanding the challenges and breakthroughs that impact sustainability in the industry and ultimately on how best to move forward with improving sustainability in surgical practice. Additional stakeholders have been identified as essential in the process of implementing sustainable practices in the surgical environment, including a broader array of drug and device manufacturers, educators, professional societies such as Surgery, Anesthesia, and Nursing societies, regulatory agencies, supply chain industry, and hospital administrators (Fig. 2).

## The importance of green operating rooms

**Fig. 3:**
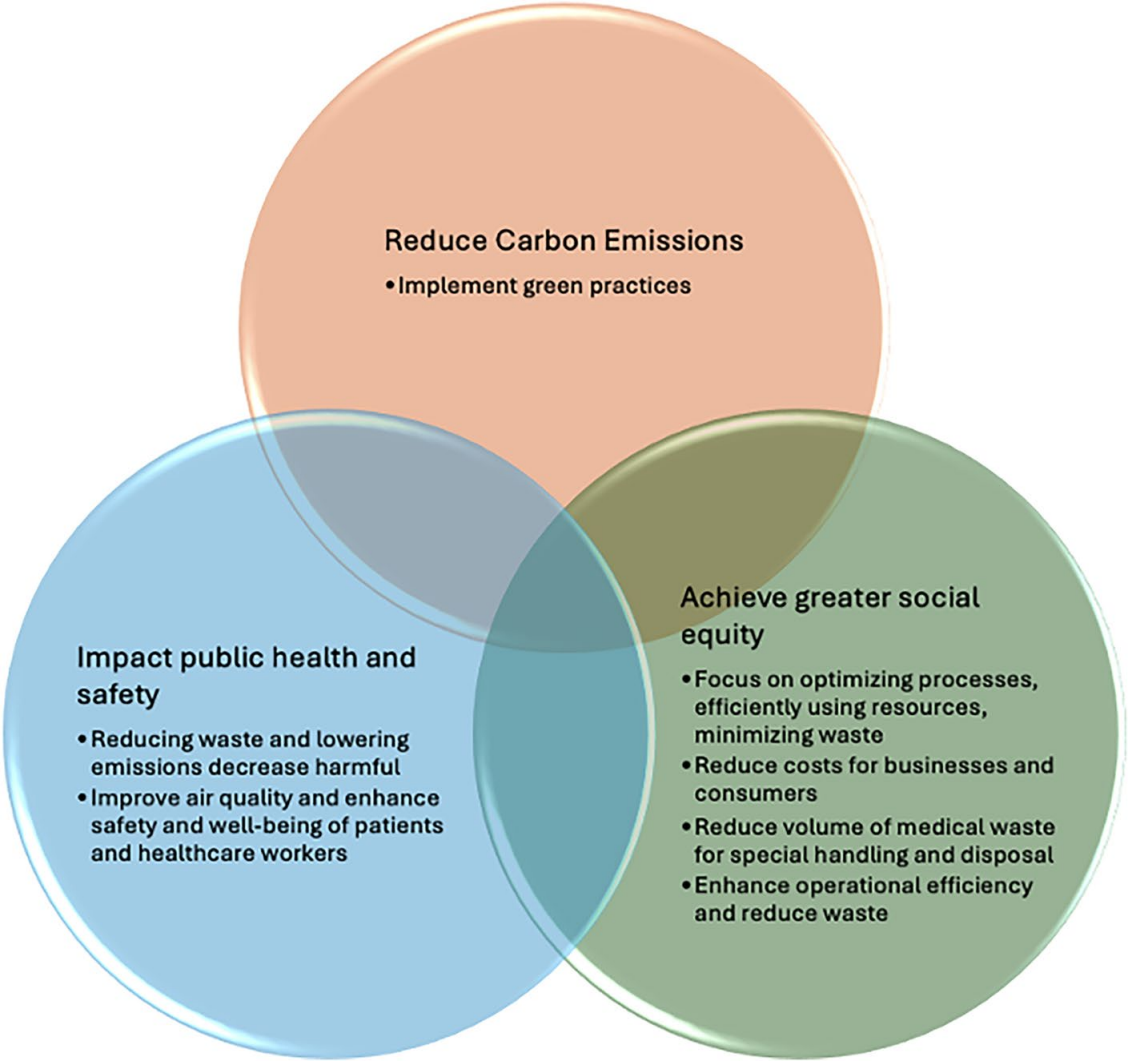
Benefits of a Green Operating Room

Rapid technological advancements and constant scientific improvements have been the norm for many decades. However, the concept of sustainability has gained importance only recently but has become equally significant. Healthcare, including surgery, is not exempt from the need for sustainability and surgical climate leadership. The development of Green Operating Rooms represents a significant shift toward responsible, ethical, and effective medical practices. Green ORs are crucial for promoting sustainable healthcare (Fig. 3). ORs generate significant greenhouse gas emissions and waste, making them a priority for implementing green practices. Adopting energy-efficient technologies and waste-reduction strategies can significantly lower utility bills, reduce costs, reduce carbon emissions, promote social equity, and align with the broader healthcare mission of promoting health and well-being [31, 32]. Sustainable OR practices can also lead to public health and safety benefits by reducing harmful pollutants and improving indoor air quality which disproportionately impacts lower income and minority communities. Embracing sustainability and Green OR practices align with consumer preferences and future-proofs businesses in a rapidly changing world.

## The role of societies and surgeons in optimizing and achieving sustainable surgical practices

Surgical societies such as EAES and SAGES, which together represent over 10,000 members with primary minimally invasive and endoscopic surgical practice, play a significant role in advancing sustainable surgical practices. First, they have a significant duty to educate surgeons and surgical teams on the environmental impact of surgical practice. This is particularly relevant to the elective setting and as it relates to the increased adoption of minimally invasive surgery (MIS) technologies, including laparoscopy and robotic surgery, alongside an unprecedented reliance on single-use equipment. Knowledge about the various elements of “carbon hotspots” in the OR can empower surgical teams to modify behavior and make incremental changes to reduce the carbon footprint without adverse consequences on patient care. This entails updating the members with the relative contribution of electricity, anesthetic agents, procedural approach (open vs. laparoscopy vs. robotic), reusable vs. disposable supplies and instruments (including procurement), and surgical waste to the overall procedural footprint. Societies may develop educational materials, guidelines, or recommendations or partner with other societies and stakeholders to disseminate best practices. Surgical societies that actively engage in research and innovation may promote, fund, and disseminate research that generates evidence to support formal recommendations and inform practice change. Lastly, surgical societies must collaborate and join global advocacy efforts alongside other stakeholders to endorse policies that bolster climate actions and increase climate resilience across the health sector.

The intersection of upholding our educational commitments towards our members while minimizing our environmental impact has created unprecedented challenges – and opportunities – for both societies. Embracing these trends can help us stay ahead of the curve and focus on promoting sustainability in all our functions.

**Fig. 4:**
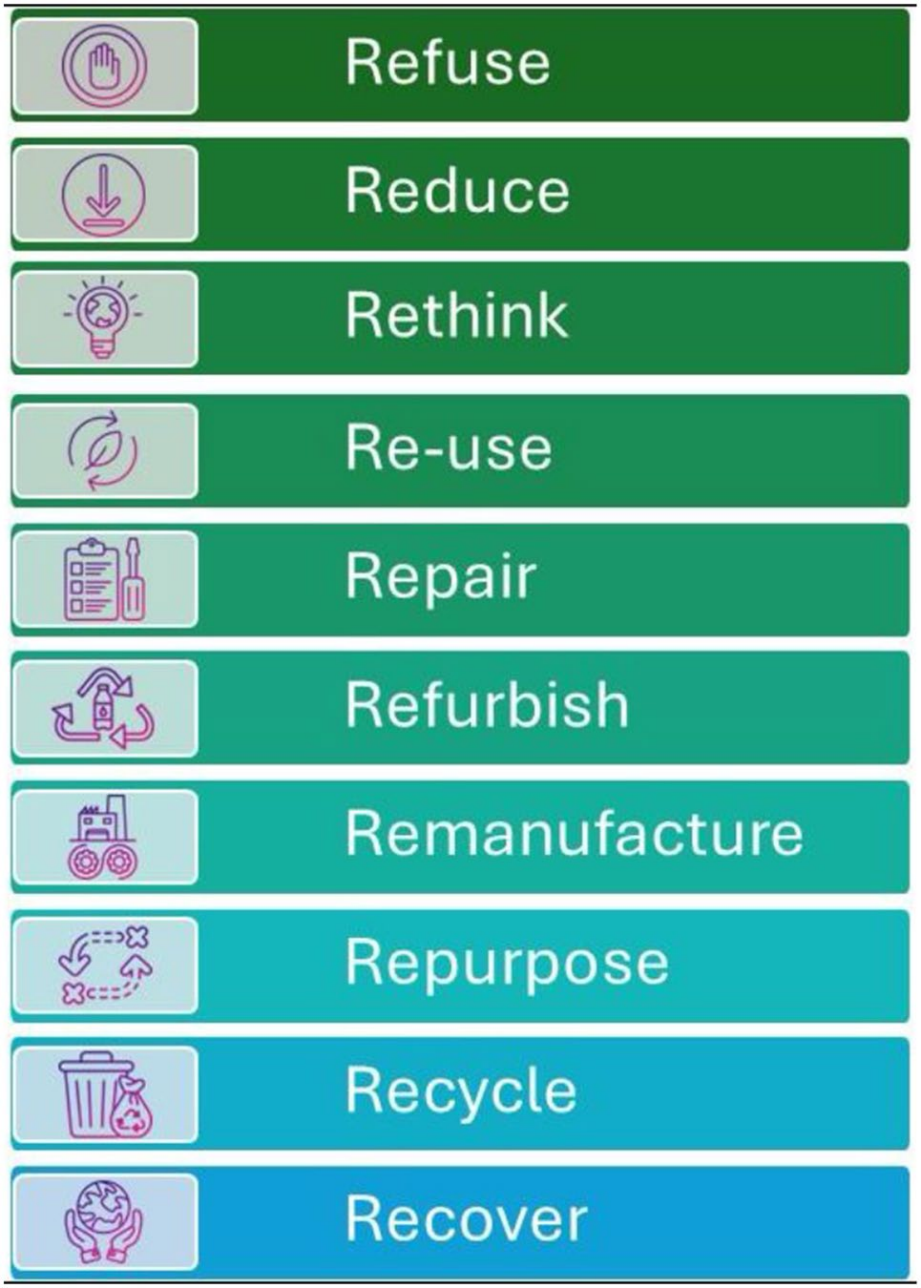
10 R model of circular economy

**Fig. 5:**
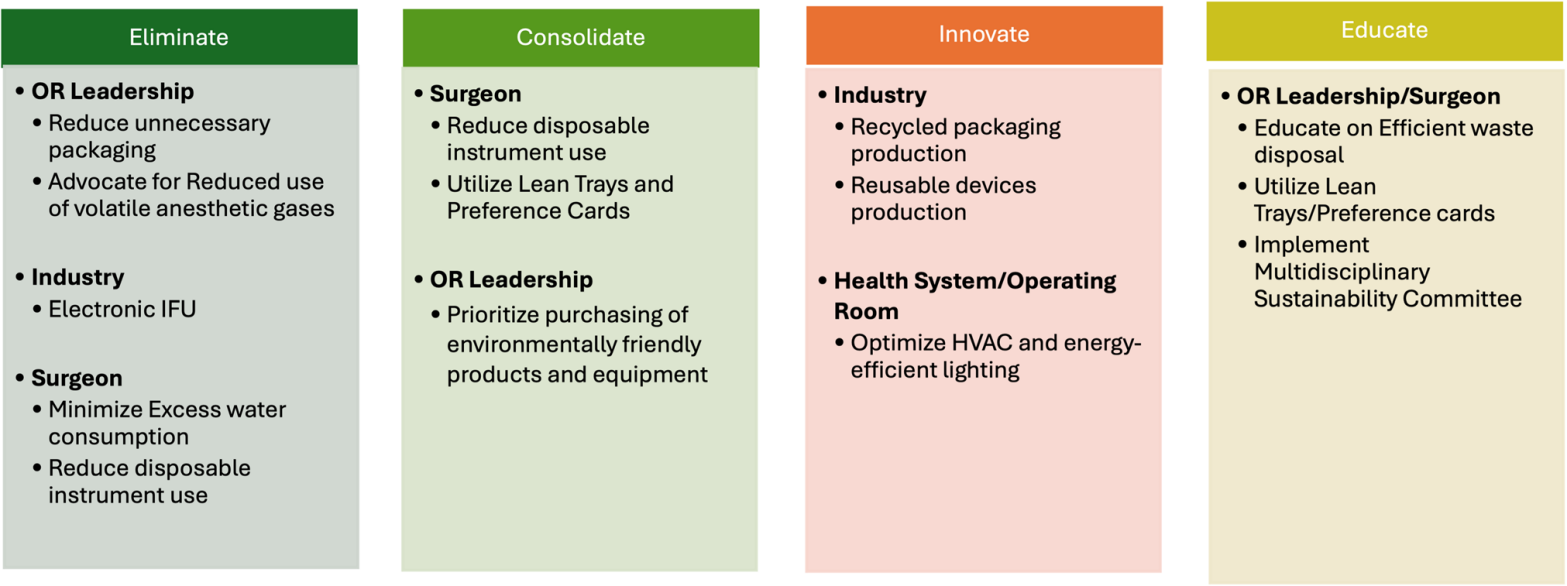
Recommendations for Initiatives to decrease carbon footprint in surgical practices

**Fig. 6:**
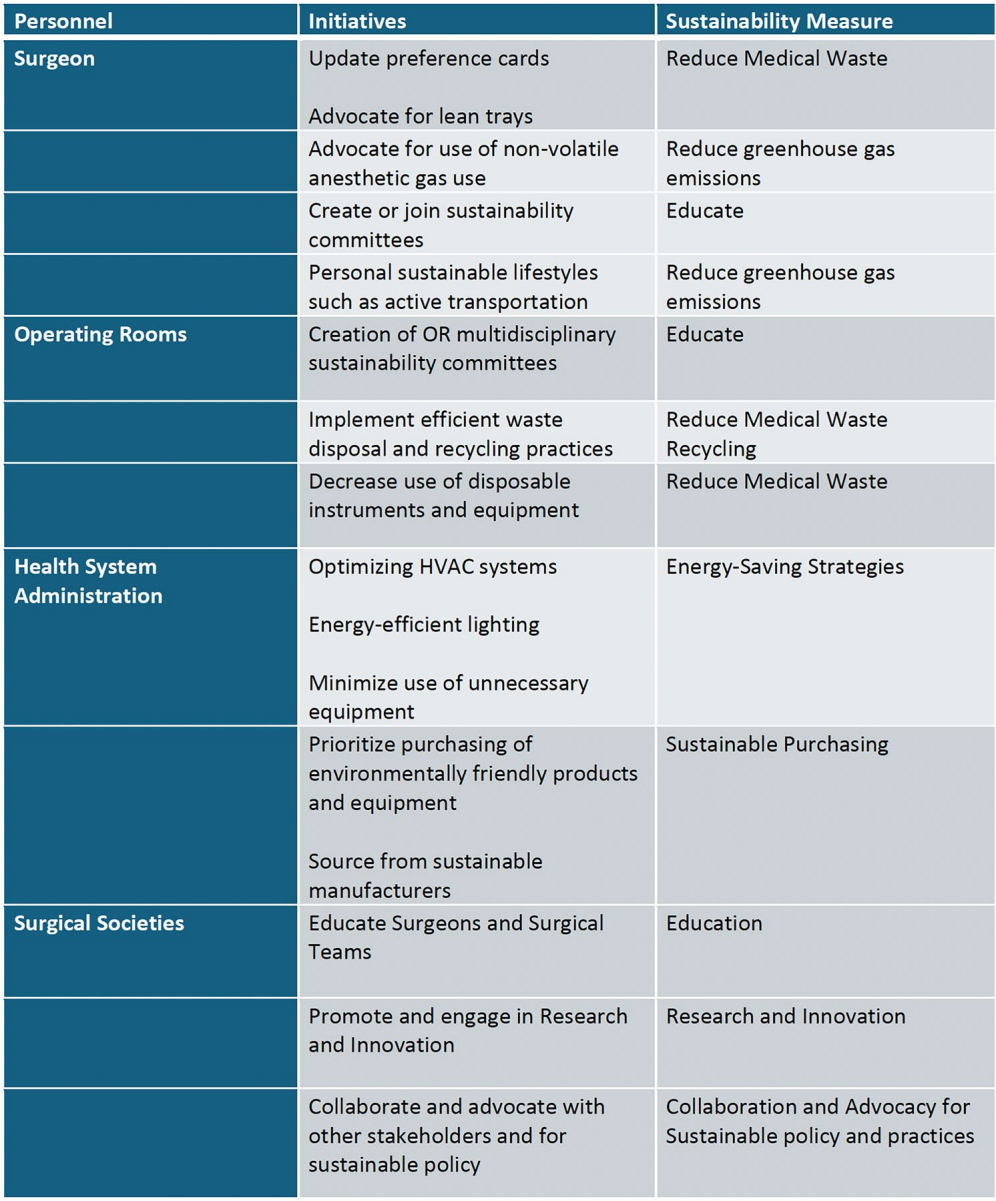
Strategies to reduce carbon footprint

A recent survey of members of the colorectal surgery community highlights the strong willingness to change current surgical practice to improve sustainability in surgery and to participate in educational webinars on sustainable actions and practices [33]. Surgeons can form or join multidisciplinary “green OR” teams at their institutions and participate in and lead changes in the operating room. Specific interventions in the operating room with measurable impacts in reducing carbon hotspots include reducing surgical waste, minimizing the use of disposable equipment, preferential use of reusable instruments, and segregating waste more effectively to promote recycling and waste disposal. Other interventions include reducing the use of inhalation anesthetic agents and promoting OR efficiency and perioperative workflow that minimize energy expenditure. The 10R model of circular economy, endorsed by the joint SSP SAGES and EAES taskforce, is an effective approach to address surgical sustainability (Fig. 4.). It aims to minimize waste, reduce costs, and enhance environmental performance while maintaining high-quality patient care. Lastly, surgeons may opt for more sustainable lifestyles and modes of transportation and virtual alternatives to in-person travel to meetings and conferences, which have been shown to generate substantial waste and carbon emissions from air and ground transportation every year [34]. The actions of surgeons, surgical practices, health system leadership, and industry via initiatives aimed at improving surgical sustainability can have a lasting impact on the carbon footprint of the healthcare system (Figs. 5, 6).

## Leading sustainability in surgical practice: a call to action

The operating room is essential for patient care but exacts a significant toll on the environment. From energy consumption to waste generation, every surgical intervention contributes to greenhouse gas emissions and resource depletion. Recognizing this, healthcare institutions must prioritize environmental sustainability as a fundamental responsibility. Surgeons, health care providers, anesthesiologists, health system administrators, societies, and regulatory agencies must work collaboratively to facilitate the dissemination of impactful, evidence-based initiatives. Surgical societies must play a central role as a repository for resources, education, and dissemination of evidence-based best practices. Whether in resource-rich or resource-constrained settings, the principles of environmental responsibility apply universally. Collaborating across borders, sharing best practices, and learning from diverse contexts will accelerate progress. The operating room represents both a challenge and an opportunity. As stewards of health and the environment, we must embrace a paradigm shift to ensure the sustainability of our practices and the planet (Table 1).

**Table 1:** Glossary of core terminology for sustainability in gastrointestinal surgical practice

Terms	Definitions
*Principles*	
Circular economy	A model of economy that involves activities that are restorative or regenerative by design and aims for the elimination of waste through the superior design of materials, products, and systems. [40]
Climate change	Shifts in weather and climate patterns that occur over long periods of time, acknowledging that the current warming temperature is caused primarily by human activity
Decarbonize	The act of reducing the amount of GHG emissions associated with a process or product, with the goal of being net neutral
Green	A colloquial term to refer to initiatives, products or practices that have environmental benefits such as reduced use of environmental resources
Greenhouse gases	Gases (primarily CO2 but including CH4, N2O, and others) that absorb, trap, and re-emit heat and radiant energy back into the earth's atmosphere1
Planetary health	An emerging concept that prioritizes solutions that simultaneously benefit human health and advance environmental sustainability. [40]
Sustainability	Meeting the needs of the present without compromising the ability of future generations to meet their own (UN Brundtland Commission) [42]
*Study designs*	
Life Cycle Assessment (LCA)	Life cycle assessments are a rigorous methodology for studying the environmental impact of a product or process. LCAs consider both the upstream and disposal of products to capture all inputs and outputs. Outcomes measured LCAs include a comprehensive range of metrics. While time intensive, these are the gold standard for sustainability studies
*Outcome measures*	
Carbon dioxide equivalents (CO2eq)	A measure of the carbon dioxide required to generate a corresponding amount of climate impact, which allows for comparison
Ecosystem quality (PDF*m2*yr or species.yr)	Potentially disappeared fraction of species in the same area, annually: a measure of the impact of climate change on the loss of biodiversity and/or species in the environment
Energy (kWh)	A measure of the amount of energy used to deliver surgical care. Since most energy currently comes from the combustion of fossil fuels, studies that show reduced energy use function as a proxy of reduced emissions
Human health (DALY/person/yr)	A measure of the impact of climate change on human health in disabilityadjusted-life-years
Particulate matter (μg/m^3^)	Particulate matter, especially that < 2.5 μm in diameter, has multi-system health risks,
Waste (kg)	A measure of the amount of waste generated to deliver surgical care
Water (m^3^)	A measure of the amount of water used to deliver surgical care
